# The International Soft Tissue Sarcoma Consortium: The baseline analysis of rhabdomyosarcoma data

**DOI:** 10.1002/cncr.35974

**Published:** 2025-07-09

**Authors:** Gianni Bisogno, Monika Sparber‐Sauer, David Rodeberg, Johannes H. M. Merks, Ewa Koscielniak, Suzanne L. Wolden, Gian Luca De Salvo, Paola Del Bianco, Wei Xue, Martin Ebinger, Christian Vokuhl, Rajkumar Venkatramani, Samuel L. Volchenboum, Brian Furner, Veronique Minard Colin, Douglas S. Hawkins

**Affiliations:** ^1^ Department of Women’s and Children’s Health University of Padua Padua Italy; ^2^ Pediatric Hematology Oncology Division University Hospital of Padua Padua Italy; ^3^ Department of Pediatric Hematology and Oncology University Children´s Hospital Tuebingen Tuebingen Germany; ^4^ Stuttgart Cancer Center Zentrum für Kinder‐, Jugend‐ und Frauenmedizin (Olgahospital) Pädiatrie 5 (Pädiatrische Onkologie, Hämatologie, Immunologie) Klinikum der Landeshauptstadt Stuttgart Stuttgart Germany; ^5^ Division of Pediatric Surgery Kentucky Children’s Hospital University of Kentucky Lexington Kentucky USA; ^6^ Princess Máxima Center for Pediatric Oncology Utrecht The Netherlands; ^7^ Division of Imaging and Oncology University Medical Center Utrecht Utrecht University Utrecht The Netherlands; ^8^ Department of Radiation Oncology Memorial Sloan Kettering New York New York USA; ^9^ Clinical Research Unit Veneto Institute of Oncology IOV‐IRCCS Padua Italy; ^10^ Department of Biostatistics University of Florida Gainesville Florida USA; ^11^ Section of Pediatric Pathology Department of Pathology University Hospital Bonn Bonn Germany; ^12^ Department of Pediatrics Baylor College of Medicine Houston Texas USA; ^13^ Department of Pediatrics University of Chicago Chicago Illinois USA; ^14^ Data for the Common Good Biological Sciences Division University of Chicago Chicago Illinois USA; ^15^ Department of Pediatric and Adolescent Oncology Gustave Roussy Université Paris‐Saclay Villejuif France; ^16^ Department of Pediatrics Seattle Children’s Hospital University of Washington Seattle Washington USA

**Keywords:** INSTRuCT, International Soft Tissue Sarcoma Consortium, Pediatric Cancer Data Commons, rhabdomyosarcoma

## Abstract

**Background:**

The International Soft Tissue Sarcoma Consortium (INSTRuCT) was established in 2017 to enhance international collaboration. This study describes the characteristics of rhabdomyosarcoma (RMS) patients in the INSTRuCT database and examines differences across contributing groups.

**Methods:**

INSTRuCT includes data Children’s Oncology Group (COG), Cooperative Weichteilsarkom Studiengruppe (CWS), and European Paediatric Soft Tissue Sarcoma Study Group (EpSSG) along with prior studies from Malignant Mesenchymal Tumour Committee (MMT) and Italian Soft Tissue Sarcoma Committee (STSC). Data standardization was supported by the University of Chicago’s Pediatric Cancer Data Commons. Pseudonymized patient‐level data from clinical trials were harmonized. Differences across groups were assessed using χ^2^ or Kruskal–Wallis tests.

**Results:**

As of March 2025, INSTRuCT includes 6972 RMS patients from 16 trials (1990–2016). Embryonal RMS was the most common histology in all groups (range, 45.4%–62.2%). Alveolar RMS was less frequent in EpSSG (26.8%) although the rate of RMS fusion‐positive was comparable across groups (74.6%–81.9%). COG and EpSSG had more T1 tumors, (53.2% and 51.4%) with COG reporting more tumors <5 cm (52%). Nodal involvement was least reported in MMT (15.4%). Metastatic patients were less represented in MMT (11%) and EpSSG (13.3%). Tumor site distribution varied: genitourinary nonbladder/prostate RMS was more common in COG, whereas head and neck nonparameningeal and orbital RMS were more represented in MMT and STSC. MMT had fewer completely resected tumors (8.9%).

**Conclusion:**

Differences among RMS study populations reflect evolving diagnostic criteria and treatment strategies that should be considered in future analyses. INSTRuCT offers a valuable international data set for RMS research.

## Abstract 2

The analysis of data from 6972 patients with rhabdomyosarcoma across 16 trials included in the International Soft Tissue Sarcoma Consortium highlights differences across the studied populations while also underscoring the potential for international collaboration.

## INTRODUCTION

Soft tissue sarcomas (STS) in children are rare cancers accounting for approximately 7%–8% of all pediatric cancers. Approximately half of them are rhabdomyosarcoma (RMS) whereas the other half consist of a variety of subtypes, ranging from the most common synovial sarcoma to very rare adult‐type STS such as leiomyosarcoma, liposarcoma, and others. The rarity of pediatric STS as a group and the different clinical and biological characteristics of the subtypes have stimulated the creation of national and international cooperative groups that have conducted a series of clinical trials that have explored different multidisciplinary treatments.^1^ Over the years, these trials have improved the prognosis of STS patients but there are still subgroups not curable with current treatments.

International collaboration between cooperative groups with clinical trial data exchange and meetings has existed since the initial trials that started in the 1970s in North America and Europe. An important step to increase research capacity has been pooling data from the different trials. These studies had precise but limited objectives.^2^, ^3^, ^4^ With the aim of creating a more comprehensive organizational structure that could be used for multiple objectives, the North American and European cooperative groups founded the International Soft Tissue Sarcoma Consortium (INSTRuCT) in 2017 and created a common data set.^5^ INSTRuCT is part of the Pediatric Cancer Data Commons run by Data for the Common Good at the University of Chicago and the process to form it has been already described.^6^

The aim of this article is to describe the characteristics of patients with RMS in the INSTRuCT database and discuss possible differences in the data contributed by the cooperative groups. This is intended to provide background for future analyses that will use INSTRuCT data.

## MATERIALS AND METHODS

INSTRuCT was established in 2017 by the predominantly North American cooperative group, Children’s Oncology Group (COG), the European cooperative groups European paediatric Soft tissue sarcoma Study Group (EpSSG), and Cooperative Weichteilsarkom Studiengruppe (CWS) and includes data from prior studies sponsored by the International Society of Paediatric Oncology (SIOP) Malignant Mesenchymal Tumour Committee (MMT) and Italian Association of Pediatric Hematology and Oncology (AIEOP) Soft Tissue Sarcoma Committee (STSC). The Pediatric Cancer Data Commons (PCDC) stewarded the process and created the procedures for aggregating and harmonizing data from different studies (Table 1).

**TABLE 1:** Studies contributing data to INSTRuCT.

Cooperative group	Studies contributing data	Period of patients enrollment	No. of patients	Total cases received (%)
COG	ARST08P1 (M)	2010–2013	167	2157 (31)
	ARST0431 (M)	2006–2008	107	
	ARST0531 (L)	2006–2012	448	
	ARST0331 (L)	2004–2011	338	
	D9802 (M)	1999–2004	108	
	D9803 (L and M)	1999–2005	597	
	D9602 (L)	1997–2004	392	
EpSSG	MTS2008 (M)	2008–2016	270	2004 (29)
	RMS2005 (L)	2005–2016	1734	
AIEOP STSC	RMS4.99 (M)	1999–2006	70	361 (5)
	RMS96 (L)	1996–2004	291	
CWS	CWS2002P (L)	2003–2010	415	1297 (19)
	CWS‐IV‐2002 (M)	2002–2010	25	
	CWS96 (L and M)	1995–2004	535	
	CWS91 (L)	1990–1995	322	
SIOP MMT	MMT95 (L and M)	1995–2003	1153	1153 (17)
Total				6972 (100)

Abbreviations: AIEOP STSC, Italian Association of Pediatric Hematology and Oncology Soft Tissue Sarcoma Committee; COG, Children’s Oncology Group; CWS, Cooperative Weichteilsarkom Studiengruppe; EpSSG, European paediatric Soft tissue sarcoma Study Group; INSTRuCT, International Soft Tissue Sarcoma Consortium; L, localized rhabdomyosarcoma; M, metastatic rhabdomyosarcoma; SIOP MMT International Society of Paediatric Oncology Malignant Mesenchymal Tumour Committee.

MMT95, CWS96, and RMS96 studies enrolled patients 0–18 years old and used the same stratification, including patients in four risk groups: low, standard, high, and metastatic (Table S1). All three studies adopted the same treatment for low and standard risk patients. In brief, low risk patients received an alkylating free regimen with vincristine and actinomycin D, standard risk patients were treated with nine cycles of ifosfamide, vincristine, and actinomycin D (IVA). In MMT95, high‐risk patients were included in a randomized trial comparing the standard IVA to the carboplatin, epirubicin, vincristine, actinomycin‐D, ifosfamide, and etoposide (CEVAIE) regimen.^7^

STSC contributed data from the RMS96 and the RMS4.99 studies. The RMS96 study was conducted in collaboration with the CWS96 trial and enrolled patients with localized disease. In high‐risk patients, the main objective was a randomized comparison of the vincristine, adriamycin, ifosfamide, and actinomycin D (VAIA) versus the CEVAIE regimen in the high‐risk population.^8^ In the RMS4.99 study, patients with metastatic RMS were included and received an intensive treatment with high‐dose sequential chemotherapy.^9^

In 2004, the MMT and STSC groups joined and founded the EpSSG and in 2005 they launched their first common protocol for localized RMS named RMS 2005.^10^ This study included observational trials for low, standard, and very high‐risk patients and two consecutive randomized trials for high‐risk patients to investigate: 1) the benefit of doxorubicin by randomizing patients to receive IVA or IVA + doxorubicin (IVADo) in the initial part of treatment; and 2) the value of maintenance chemotherapy for patients in complete remission after standard therapy by adding six 4‐week long cycles of vinorelbine and low dose oral cyclophosphamide.

EpSSG also contributed to INSTRUCT with the MTS 2008 study that enrolled metastatic patients and treated them with nine cycles of chemotherapy (four IVADo + five IVA) followed by 1 year of maintenance with vinorelbine and low dose cyclophosphamide.^11^ Selected EpSSG centers enrolled metastatic patients also in the parallel Bernie Study (2008–2013) a pharmaceutical company sponsored trial of whose data have not yet been transferred into the INSTRuCT database.^12^

CWS included data from the CWS‐91, CWS‐96, and CWS‐2002P studies that enrolled patients with localized disease. In high‐risk patients, the VAIA combination was used as standard chemotherapy.^8^, ^13^ In CWS‐2002P, maintenance chemotherapy with low dose cyclophosphamide and vinblastine was added.^14^

The CWS‐IV‐2002 study enrolled patients with metastatic disease. Patients were treated with the VAIA or CEVAIA regimens followed by maintenance chemotherapy with trofosfamide and etoposide alternating with trofosfamide and idarubicin or low dose cyclophosphamide and vinblastine. A minority of them underwent allogeneic hematopoietic stem cell transplantation.^15^

COG included data from seven different studies conducted between 1997 and 2013 for low‐risk (D9602 and ARST0331), intermediate‐risk (D9803 and ARST0531), and high‐risk (D9802, ARST0431, and ARST08P1) RMS. The backbone of all these studies was the vincristine, actinomycin D, and cyclophosphamide regimen. In brief, low‐risk trials sought to optimize outcome with no or limited dose cyclophosphamide using single arms with historical controls; intermediate‐risk trials sought to improve outcome with the randomized addition of topotecan or irinotecan with concurrent controls; and high‐risk trials sought to improve outcome with the addition of irinotecan, intensified multi‐agent chemotherapy, or the addition of temozolomide or cixutumumab using single arms with historical controls.^16^, ^17^, ^18^, ^19^, ^20^, ^21^, ^22^

A total of 6972 patients with RMS have been registered in INSTRuCT from participating cooperative groups creating a standardized data dictionary. Pseudonymized patient data were used removing all patient identifiers and assigning pseudonymized identification numbers by an honest broker as previously described.^6^

Histology is reported here as initially registered in different studies and later grouped according to the prognosis with unfavorable histology including alveolar RMS (ARMS), mixed (alveolar and embryonal), and RMS not otherwise specified. All other subtypes were included in the favorable category but pleomorphic RMS were grouped separately

Primary tumor invasiveness, nodal involvement, and presence of metastasis were categorized according to the TNM staging system: T1 and T2 indicate tumors without or with invasion into adjacent organs, respectively; N0 and N1 represent the absence or presence of regional lymph node involvement; and M0 and M1 denote the absence or presence of distant metastases at diagnosis.

The tumor was also classified according to the Intergroup Rhabdomyosarcoma Study (IRS) clinical grouping system, which categorizes patients based on the extent of disease after initial surgery: group I includes localized tumors completely resected with no residual disease; group II includes tumors with microscopic residual disease or regional lymph node involvement; group III includes tumors with gross residual disease after incomplete resection or biopsy; and group IV includes patients with distant metastases at diagnosis.

Because the data were collected over a long period in which diagnostic criteria, classification system, and biological markers may have changed,^23^ we also compared the early versus the late generation of trials comparing those that started before or after 2003.

Summary statistics and analyses are presented by cooperative groups. Categorical variables are summarized as frequencies and percentages. Descriptive statistics for continuous variables include median, first quartile (Q1) and third quartile (Q3). R version 4.2.2 was used for all data summaries and statistical analyses. A comparison of the data contributed by the different cooperative groups has been made and differences among the data contributed were compared using χ^2^ or Kruskal–Wallis test as appropriate.

No survival data are presented in this article as they will be part of specific analyses.

## RESULTS

This analysis includes data from 6972 patients with RMS, of whom 30.9% were registered in COG studies, with the remaining patients enrolled in various European studies (Table 1). Regarding the enrollment period, trials opened before or after 2003 included 3360 patients (48.2%) and 3612 patients (51.8%), respectively.

The data set is 93% complete, with no missing values for histology, invasiveness, and nodal involvement, and minimal missing values in all other variables (affecting at most 4% of records) (Figure S1).

The distribution of clinical characteristics differed significantly across the data sets provided by the various cooperative groups for all variables (*p* < .001), with the exception of age, which did not show a statistically significant difference (*p* = .075). The main differences are reported below.

Across all groups, males outnumbered females, with the proportion of males ranging from 57% in CWS to 61.3% in MMT (Table 2). The median age was relatively consistent across groups, varying from 5.4 years (MMT) to 6.5 years (COG). European studies had the higher proportion of patients under 1 year of age and COG had the most >18 years of age (6.3%). In total, 281 patients over 18 years of age were enrolled, with 216 of them (77%) participating in the protocols open after 2003.

**TABLE 2:** Patients’ characteristics by cooperative groups.

	COG (*N* = 2157)	CWS (*N* = 1297)	EpSSG (*N* = 2004)	AIEOP STSC (*N* = 361)	SIOP MMT (*N* = 1153)	Total (*N* = 6972)
Era						
≤2003	989 (45.9%)	857 (66.1%)	0 (0.0%)	361 (100.0%)	1153 (100.0%)	3360 (48.2%)
>2003	1168 (54.1%)	440 (33.9%)	2004 (100.0%)	0 (0.0%)	0 (0.0%)	3612 (51.8%)
Sex						
Female	858 (39.8%)	558 (43.0%)	803 (40.1%)	144 (39.9%)	445 (38.7%)	2808 (40.3%)
Male	1299 (60.2%)	739 (57.0%)	1201 (59.9%)	217 (60.1%)	705 (61.3%)	4161 (59.7%)
Missing					3	3
Age (years)						
Median (range)	6.5 (0.0, 45.7)	5.9 (0.0, 20.8)	5.6 (0.0, 25.0)	5.6 (0.0, 20.9)	5.4 (0.0, 32.5)	5.9 (0.0, 45.7)
<1	95 (4.4%)	84 (6.5%)	113 (5.6%)	24 (6.6%)	76 (6.6%)	392 (5.6%)
1–3	404 (18.7%)	220 (17.0%)	415 (20.7%)	66 (18.3%)	221 (19.3%)	1326 (19.0%)
3–10	867 (40.2%)	591 (45.6%)	900 (44.9%)	165 (45.7%)	574 (50.0%)	3097 (44.5%)
10–18	656 (30.4%)	358 (27.6%)	493 (24.6%)	101 (28.0%)	263 (22.9%)	1871 (26.9%)
>18	135 (6.3%)	44 (3.4%)	83 (4.1%)	5 (1.4%)	14 (1.2%)	281 (4.0%)
Missing					5	5
Histology subtypes^a^						
Spindle cell	180 (8.3%)	13 (1.0%)	72 (3.6%)	5 (1.4%)	0 (0.0%)	270 (3.9%)
Botryoid	205 (9.5%)	64 (4.9%)	101 (5.0%)	25 (6.9%)	91 (7.9%)	486 (7.0%)
Embryonal	979 (45.4%)	794 (62.2%)	1246 (62.2%)	203 (56.2%)	574 (49.8%)	3796 (54.7%)
Alveolar	688 (31.9%)	364 (28.0%)	538 (26.8%)	121 (33.5%)	403 (35.0%)	2114 (30.5%)
Mixed	20 (0.9%)	1 (0.1%)	19 (0.9%)	0 (0.0%)	38 (3.3%)	78 (1.1%)
NOS	85 (3.9%)	46 (3.5%)	27 (1.3%)	5 (1.4%)	0 (0.0%)	132 (1.9%)
Pleomorphic	0 (0.0%)	15 (1.1%)	1 (0.0%)	2 (0.6%)	47 (4.1%)	65 (0.9%)
Histology by prognosis^b^						
Favorable	1364 (63.2%)	871 (67.1%)	1419 (70.8%)	233 (64.5%)	665 (57.7%)	4552 (65.6%)
Unfavorable	793 (36.7%)	411 (31.7%)	584 (29.1%)	126 (34.9%)	441 (38.2%)	2324 (33.5%)
Pleomorphic	0 (0.0%)	15 (1.2%)	1 (0.0%)	2 (0.6%)	47 (4.1%)	65 (0.9%)
Anaplasia						
Present	351 (17.9%)		235 (16.7%)			586 (17.4%)
Absent	1615 (82.1%)		1171 (83.3%)			2786 (82.6%)
Missing	191	1297	598	361	1153	3600
PAX3/7‐FOXO1						
Positive	409 (68.1%)	158 (53.4%)	379 (24.9%)	39 (62.9%)	49 (29.7%)	1034 (39.1%)
Negative	192 (31.9%)	138 (46.6%)	1143 (75.1%)	23 (37.1%)	116 (70.3%)	1612 (60.9%)
Not assessed	1556	1001	482	299	988	4326
Invasiveness						
T1	1148 (53.2%)	416 (32.1%)	1030 (51.4%)	159 (44.0%)	433 (37.6%)	3186 (45.7%)
T2	1004 (46.5%)	804 (62.0%)	957 (47.8%)	197 (54.6%)	645 (55.9%)	3607 (51.7%)
TX	5 (0.2%)	77 (5.9%)	17 (0.8%)	5 (1.4%)	75 (6.5%)	179 (2.6%)
Nodal involvement						
N0	1662 (77.1%)	835 (64.4%)	1539 (76.8%)	281 (77.8%)	869 (75.4%)	5186 (74.4%)
N1	465 (21.6%)	323 (24.9%)	452 (22.6%)	78 (21.6%)	178 (15.4%)	1496 (21.5%)
NX	30 (1.4%)	139 (10.7%)	13 (0.6%)	2 (0.6%)	106 (9.2%)	290 (4.2%)
Metastasis						
M0	1724 (80.0%)	863 (75.0%)	1734 (86.5%)	291 (80.6%)	985 (89.0%)	5597 (82.6%)
M1	430 (20.0%)	288 (25.0%)	270 (13.5%)	70 (19.4%)	122 (11.0%)	1180 (17.4%)
Missing	3	146			46	195
Tumor size						
Median (range)	5.0 (0.1, 50.2)	‐‐	5.4 (0.2, 34.0)	5.7 (0.6, 30.0)	5.3 (0.2, 24.0)	5.2 (0.1, 50.2)
≤5 cm	1101 (52.0%)	521 (41.9%)	892 (45.1%)	153 (43.3%)	481 (48.4%)	3148 (47.1%)
>5 cm	1016 (48.0%)	722 (58.1%)	1087 (54.9%)	200 (56.7%)	512 (51.6%)	3537 (52.9%)
Missing	40	54	25	8	160	287
Tumor site						
Orbit	218 (10.3%)	103 (8.1%)	186 (9.3%)	35 (9.7%)	137 (12.0%)	679 (9.8%)
HNnoPM	179 (8.4%)	90 (7.1%)	174 (8.7%)	36 (10.0%)	201 (17.6%)	680 (9.8%)
HNPM	433 (20.4%)	290 (22.7%)	475 (23.7%)	61 (16.9%)	174 (15.3%)	1433 (20.8%)
GUBP	193 (9.1%)	133 (10.4%)	229 (11.4%)	33 (9.1%)	108 (9.5%)	696 (10.1%)
GUnoBP	430 (20.2%)	188 (14.7%)	354 (17.7%)	66 (18.3%)	160 (14.0%)	1198 (17.4%)
Extremity	265 (12.5%)	189 (14.8%)	265 (13.2%)	50 (13.9%)	123 (10.8%)	892 (12.9%)
Other	407 (19.2%)	282 (22.1%)	321 (16.0%)	80 (22.2%)	236 (20.7%)	1326 (19.2%)
Missing	32	22			14	68
IRS group						
Group I	319 (14.8%)	148 (12.9%)	212 (10.6%)	53 (14.7%)	98 (8.9%)	830 (12.2%)
Group II	337 (15.6%)	140 (12.2%)	216 (10.8%)	50 (13.9%)	134 (12.1%)	877 (12.9%)
Group III	1068 (49.6%)	575 (50.0%)	1306 (65.2%)	188 (52.1%)	753 (68.0%)	3890 (57.4%)
Group IV	430 (20.0%)	288 (25.0%)	270 (13.5%)	70 (19.4%)	122 (11.0%)	1180 (17.4%)
Missing	3	146			46	195

*Note*: Clinical characteristics distribution among the data contributed by the different cooperative groups resulted statistically significant different for all variables (*p* < .001) apart from age (*p =* 0.075).

Abbreviations: AIEOP STSC Italian Association of Pediatric Hematology and Oncology Soft Tissue Sarcoma Committee; COG, Children’s Oncology Group; CWS, Cooperative Weichteilsarkom Studiengruppe; EpSSG, European paediatric Soft tissue sarcoma Study Group; GUBP, genitourinary bladder prostate; GUnoBP, genitourinary nonbladder/prostate; HNnoPM, head and neck non parameningeal; HNPM, head and neck parameningeal; IRS, Intergroup Rhabdomyosarcoma Study; NOS, not otherwise specified; SIOP MMT, International Society of Paediatric Oncology Malignant Mesenchymal Tumour Committee.

^a^ Mixed means embryonal rhabdomyosarcoma with the presence of an alveolar component.

^b^ Favorable includes spindle cells botryoid and embryonal rhabdomyosarcoma. Unfavorable includes alveolar, mixed, and NOS rhabdomyosarcoma.

Information on race and ethnicity was not collected in the European studies and is therefore not presented here.

Histology was categorized into seven subtypes, reflecting the evolution of RMS diagnostic criteria over time. Embryonal RMS (ERMS) was the most common histology type across all groups ranging from 45.4% in COG patients to 62.2% in CWS and EpSSG. Alveolar ARMS was less frequently diagnosed in the EpSSG study (26.8%). Differences in other subtypes were observed: spindle cell RMS was more common in the COG population, whereas RMS with mixed features was more prevalent in MMT. Pleomorphic RMS patients were differently represented in the contributing studies: they were registered in the MMT and CWS protocols, considered not eligible in the EpSSG study and called anaplastic RMS in COG trials. When grouped according to prognosis, approximately one‐third of INSTRuCT patients were classified as unfavorable (alveolar, mixed, and not otherwise specified RMS). Data on anaplasia was collected only by COG and EpSSG.

The fusion gene status was often not assessed, with data reported for only 38% of patients, independently from the RMS subtype. This proportion has increased from 17.9% in earlier studies to 56.8% in more recent ones (i.e., open before and after 2003) (Tables S2 and S3). In the most recent studies, the proportion of patients with fusion gene status investigated was 45.9% in the group with favorable histology and 77.8% in the group with unfavorable histology. A variability across cooperative groups was observed, with fewer patients having their fusion status investigated in the group with favorable histology in the COG (2.8%) and CWS (19.2%) studies compared to the EpSSG study (71.7%). However, the proportion of patients with unfavorable histology and fusion status positive was consistent across all groups (Table 3), ranging from 74.6% in the EpSSG to 81.4% in the COG and 81.9% in CWS studies.

**TABLE 3:** Fusion status by cooperative group in the most recent protocols (>2003).

		COG (*N* = 1168)	CWS (*N* = 433)	EpSSG (*N* = 2003)	Total (*N* = 3604)
Histology	PAX3/7‐FOXO1				
	Assessed	364 (31.2%)	160 (37.0%)	1522 (76.0%)	2046 (56.8%)
Favorable	Positive	0 (0%)	3 (1%)	3 (0.2%)	6 (0.2%)
	Negative	19 (2.8%)	52 (18.2%)	1015 (71.5%)	1086 (45.7%)
	Not assessed	655 (97.2%)	231 (80.8%)	401 (28.3%)	1287 (54.1%)
	Total	674	286	1419	2379
Unfavorable	Positive	281 (56.9%)	86 (58.5%)	376 (64.4%)	743 (60.6%)
	Negative	64 (12.9%)	19 (12.9%)	128 (21.9%)	211 (17.2%)
	Not assessed	149 (30.2%)	42 (28.6%)	80 (13.7%)	271 (22.2%)
	Total	494	147	584	1225

Abbreviations: COG, Children’s Oncology Group; CWS, Cooperative Weichteilsarkom Studiengruppe; EpSSG, European paediatric Soft tissue sarcoma Study Group.

As expected, most patients presented with invasive (T2) and large (>5 cm) tumors. Patients enrolled in COG and EpSSG studies had a higher proportion of T1 (53.2% and 51.4%). COG also had a higher proportion of <5 cm tumors (52%). Regional nodal involvement was reported in 21% of cases in total, with a lower number of patients in the MMT study (15.4%). The proportion of patients with distant metastasis was lower in MMT (11%) and EpSSG (13.3%) studies.

Tumor site distribution was generally similar across groups, with a few notable exceptions. COG studies included a higher proportion of genitourinary nonbladder/prostate tumors (20.2%). SIOP MMT and AIEOP STSC studies reported fewer head and neck parameningeal tumors, but a higher number of head and neck nonparameningeal and orbital RMS cases (Table 2).

Post‐surgical classifications, as defined by the IRS grouping system,^24^ showed minor variations. In MMT studies, the proportion of patients with completely resected tumors was lower (8.9%) compared to other studies, whereas the number of IRS group III patients was higher (68%) and similar to that observed in EpSSG (65.2%).

## DISCUSSION

Here, we present an outline of RMS data collected by INSTRuCT. This data set has inherent limitations in epidemiological value due to the design of the contributing studies, which spanned different time periods and employed varying eligibility criteria. For instance, in Europe early studies limited the age of enrollment to 18 years and the most recent protocols included patients up to the age of 21 years whereas the age limit for all COG studies was 50 years. Another limitation is the long timeframe covered, during which diagnostic definitions and techniques have evolved introducing variability into the data set.

Differences might have been determined by the different nature of contributing studies as some of them were randomized trials with restricted eligibility criteria and others allowed the registration of all patients with a diagnosis of RMS. This is the case of the EpSSG trials that registered nearly 75% of all children and adolescents with RMS diagnosed in the participating countries.^25^

When INSTRuCT was initiated, significant effort was devoted to developing a standardized data dictionary to facilitate reliable and meaningful data collection. To make this endeavor feasible, a pragmatic approach was taken to select the most critical variables, focusing on key patient and tumor characteristics along with limited treatment‐related data. These variables can now be explored by the public using the cohort discovery tool available on the INSTRuCT website.^6^

Even with these caveats, this data set represents the largest collection of high‐quality RMS clinical trial data to date, enabling robust analyses of both the general population and specific subgroups. The analysis presented here provides critical insights into the available data, highlights notable differences across groups, and underscores gaps in data collection. For example, race and ethnicity data were collected only by COG, precluding analysis of these variables across the entire INSTRuCT data set.

Diagnostic consistency is a critical consideration in the INSTRuCT data set. Variations in histological diagnoses across cooperative groups largely reflect changes in RMS diagnostic criteria over time. These changes have been well described.^23^ For instance, MMT protocols and early COG trials adopted diagnostic criteria for alveolar RMS that included focal rather than predominant alveolar features, contributing to the higher alveolar RMS rate observed in MMT populations. Additionally, the transition from the international classification of RMS^26^ to the World Health Organization classification and the increasing use of FOXO1 fusion status to support ARMS diagnoses have further contributed to these differences.^23^

When FOXO1 fusion status is used to identify patients with unfavorable histology, as in more recent protocols, the proportion of patients classified with unfavorable diagnoses drops significantly—from 31.2% (alveolar RMS cases) to 20.9% (fusion‐positive cases). This illustrates the profound impact of integrating molecular markers into the diagnostic process. The search for PAX3/7‐FOXO1 translocation was limited in earlier trials but has become progressively more accessible in recent studies. Differences in the number of patients with known fusion status also arise from varying strategies across groups. For example, EpSSG systematically recommended FOXO1 translocation testing, whereas in COG and CWS studies, the test was done at a reference laboratory but it was not mandatory when ERMS was diagnosed.

To address the challenge of missing data, fusion status imputation has been employed in previous studies.^27^, ^28^ This choice was based on the fact that virtually all ERMS are fusion‐negative along with the need to maximize the utility of the data set while minimizing biases associated with incomplete molecular data. INSTRUCT data confirm this assumption and therefore we propose to adopt fusion status imputation in future INSTRuCT analysis.

Anaplasia, another important feature, was assessed differently among the contributing groups. For instance, the systematic collection of anaplasia data was the focus of a specific COG study,^29^ whereas in Europe, only EpSSG routinely gathered this information.

Variations in clinical characteristics of patients were also evident across groups, potentially reflecting differences in definitions and organizational aspects. For example, the lower number of metastatic patients reported in early European studies MMT and AIEOP STSC is explained by the fact that the enrollment of metastatic patients started later in these protocols that initially included only patients with localized disease.^7^, ^8^, ^9^, ^30^

Despite an increased convergence of the definition and treatment strategies used by the different international groups, differences still exist and this became immediately apparent when INSTRuCT was formed. As an initial but ongoing effort, the different discipline panels created in INSTRuCT have worked to create a set of common definitions and recommendations for RMS diagnosis and treatment. These efforts have resulted in consensus agreements that are progressively establishing a shared international language for RMS studies.^23^, ^31^, ^32^

In conclusion, this article provides an overview of the data included in INSTRuCT database and its potential to be used for future analyses. Understanding the composition of the INSTRuCT data set and the differences among populations included by the contributing RMS studies is essential for interpreting future findings. Despite the limitations discussed, INSTRuCT and its data set represent a powerful initiative, laying the groundwork for a unified international approach to RMS research.

## AUTHOR CONTRIBUTIONS

**Gianni Bisogno:** Conceptualization, writing–original draft, writing–review and editing, data curation, methodology, investigation, and supervision. **Monika Sparber‐Sauer:** Data curation, investigation, writing–review and editing, writing–original draft, validation, and supervision. **David Rodeberg:** Investigation, writing–review and editing, and validation. **Johannes H. M. Merks:** Investigation, validation, writing–review and editing, and supervision. **Ewa Koscielniak:** Investigation, validation, writing–review and editing, data curation, and supervision. **Susan L. Wolden:** Investigation, writing–review and editing, and validation. **Gian Luca De Salvo:** Investigation, formal analysis, methodology, writing–original draft, writing–review and editing, and validation. **Paola Del Bianco:** Investigation, writing–original draft, writing–review and editing, methodology, formal analysis, and validation. **Wei Xue:** Investigation, methodology, formal analysis, validation, and writing–review and editing. **Martin Ebinger:** Investigation, writing–review and editing, and validation. **Christian Vokuhl:** Investigation, methodology, validation, visualization, and writing–review and editing. **Rajkumar Venkatramani:** Investigation, writing–review and editing, validation, and data curation. **Samuel L. Volchenboum:** Supervision, investigation, validation, and writing–review and editing. **Brian Furner:** Investigation, methodology, visualization, writing–review and editing, software, and supervision. **Veronique Minard Colin:** Investigation, validation, visualization, writing–review and editing, and supervision. **Douglas S. Hawkins:** Conceptualization, investigation, methodology, validation, writing–original draft, writing–review and editing, data curation, and supervision.

## CONFLICT OF INTEREST STATEMENT

Brian Furner reports stock holdings with Cisco Systems, Microsoft Corporation, and United Therapeutics Corporation. Johannes H. M. Merks reports consulting fees from Bayer, GlaxoSmithKline, and Merck. Monika Sparber‐Sauer reports grant and/or contract funding from Deutsche Kinderkrebsstiftung and Deutsche Krebshilfe. Samuel L. Volchenboum reports consulting fees from Belay Diagnostics and CVS Accordant; and stock holdings with Litmus Health. The other authors declare no conflicts of interest.

## Supporting information

Supplementary Material

## Data Availability

The data that support the findings of this study are available on request from the corresponding author. The data are not publicly available due to privacy or ethical restrictions.
