# Supplementary material for: The International Soft Tissue Sarcoma Consortium: The baseline analysis of rhabdomyosarcoma data
Source: Cancer. 2025 Jul 9;131(14):e35974. doi: 10.1002/cncr.35974 (PMC12239856; doi:10.1002/cncr.35974)
Supplement: Supplementary file 1 — Supplementary Material [file CNCR-131-e35974-s001.docx]

Supplemental material

**Table 1 – EpSSG risk stratification for non metastatic rhabdomyosarcoma**

| **Risk Group** | **Subgroups** | **Pathology** | **Post surgical Stage**  **(IRS Group)** | **Site** | **Node**  **Stage** | **Size & Age** |
| --- | --- | --- | --- | --- | --- | --- |
| **Low Risk** | **A** | Favourable | I | Any | N0 | Favourable |
| **Standard Risk** | ***B*** | Favourable | I | Any | N0 | Unfavourable |
|  | ***C*** | Favourable | II, III | Favourable | N0 | Any |
|  | ***D*** | Favourable | II, III | Unfavourable | N0 | Favourable |
| **High Risk** | ***E*** | Favourable | II, III | Unfavourable | N0 | Unfavourable |
|  | ***F*** | Favourable | II, III | Any | N1 | Any |
|  | ***G*** | Unfavourable | I, II, III | Any | N0 | Any |
| **Very High Risk** | ***H*** | Unfavourable | I, II, III | Any | N1 | Any |

- **Pathology:**

*Favourable* = all embryonal, spindle cells, botryoid RMS

*Unfavourable* = all alveolar RMS (including the solid-alveolar variant)

- **Post surgical stage** (according to the IRS grouping, see appendix A.2):

*Group I* = primary complete resection (R0);

*Group II* = microscopic residual (R1) or primary complete resection but N1;

*Group III* = macroscopic residual (R2);

- **Site:**

*Favourable* = orbit, GU non bladder prostate (i.e. paratesticular and vagina/uterus) and non PM head & neck

*Unfavourable* = all other sites (parameningeal, extremities, GU bladder-prostate and “other site”)

- **Node stage** (According to the TNM classification, see appendix A1 and A.5):

*N0* = no clinical or pathological node involvement

*N1* = clinical or pathological nodal involvement

- **Size & Age:**

*Favourable* = Tumour size (maximum dimension) <5cm *and* Age <10 years

*Unfavourable* = all others (i.e. Size >5 cm ***or*** Age ≥10 years)

**Figure 1 – INSTRuCT Missing data pattern.**


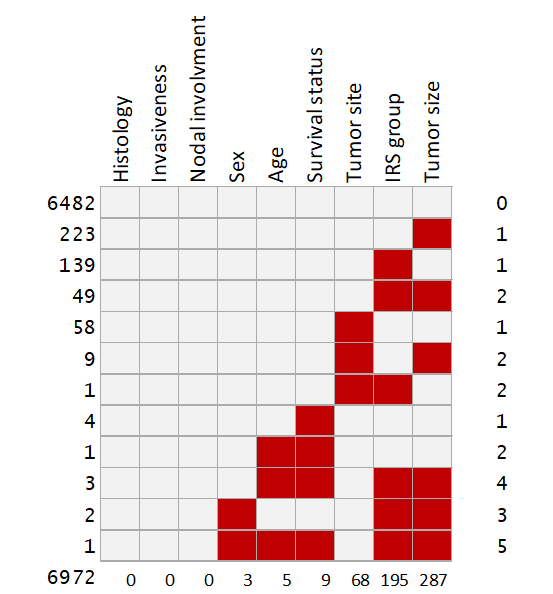


Missing data matrix where rows correspond to different missing data patterns, columns represent variables and red cells indicate the missing variable. The numbers on the left indicate the number of cases with each missing pattern (as an example: second row indicate that there are 223 patients with one variable missing (tumor size). The numbers on the right indicate the total number of missing variables for each pattern (as an example considering the last row a total of 5 variables are missing in 1 patient). The bottom row shows the total number of missing values for each variable (as an example the last column indicate that in 287 patients the variable tumor size is missing).

Data on anaplasia were collected exclusively by COG and EpSSG. Fusion status has been assessed mostly in recent years and primarily for alveolar rhabdomyosarcoma patients, therefore these variables were not considered in the missing data matrix.

# Table 2 - Fusion Status by cooperative Group in the early studies (< 2003)

|  |  | COG  (N=989) | CWS  (N=857) | AIEOP STSC (N=361) | SIOP MMT  (N=1153) | Total  (N=3360) |
| --- | --- | --- | --- | --- | --- | --- |
| Histology | PAX3/7-FOXO1 |  |  |  |  |  |
|  | Assessed | 237  (24%) | 136  (15.9%) | 62  (17.2%) | 165  (14.3%) | 600  (17.9%) |
| Favorable | Positive | 1  (0.1%) | 2  (0.2%) | 0 | 11  (0.9%) | 14  (0.4%) |
|  | Negative | 50  (5.0%) | 48  (5.6%) | 0 | 58  (5.0%) | 156  (4.6%) |
|  | Not assessed | 639 (64.6%) | 535  (62.4%) | 233  (64.5%) | 596  (51.7%) | 2003  (59.6%) |
|  | Total | 690 | 585 | 233 | 665 | 2173 |
| Unfavorable | Positive | 127 (12.8%) | 67  (7.8%) | 39  (10.8%) | 37  (3.2%) | 270  (8.0%) |
|  | Negative | 59  (6.0%) | 17  (2.0%) | 23  (6.4%) | 50  (4.3%) | 149  (4.4%) |
|  | Not assessed | 113  (11.4%) | 180  (21.0%) | 64  (17.7%) | 354  (30.7%) | 711  (21.2%) |
|  | Total | 299 | 264 | 126 | 441 | 1130 |
| Pleomorphic | Positive | 0 | 0 | 0 | 1  (0.1%) | 1  (0.03%) |
|  | Negative | 0 | 2  (0.2%) | 0 | 8  (0.7%) | 10  (0.3%) |
|  | Not assessed | 0 | 6  (0.7%) | 2  (0.5%) | 38  (3.3%) | 46  (1.4%) |
|  | Total | 0 | 8 | 2 | 47 | 57 |

COG Children’s Oncology Group, EpSSG European paediatric Soft tissue sarcoma Study Group, CWS Cooperative Weichteilsarkom Studiengruppe. SIOP MMT International Society of Paediatric Oncology Malignant Mesenchymal Tumour Committee, AIEOP STSC Italian Association of Pediatric Hematology and Oncology Soft Tissue Sarcoma Committee.
